# Osteomyelitis in peri-implant bone of hip prosthetic joint infection: prevalence and clinical impact

**DOI:** 10.5194/jbji-11-43-2026

**Published:** 2026-01-15

**Authors:** Ernesto Muñoz-Mahamud, Melissa Rivera, Ana Belén Larque, Laura Morata, Andrés Combalia, Alfonso Alías, Jenaro Ángel Fernández-Valencia, Álex Soriano

**Affiliations:** 1 Servei de Cirurgia Ortopèdica i Traumatologia, Hospital Clínic de Barcelona, Universitat de Barcelona, C/Villarroel 170, 08036 Barcelona, Spain; 2 Departament de Cirurgia i Especialitats Medicoquirúrgiques, Facultat de Medicina i Ciències de la Salut, Universitat de Barcelona (UB), Barcelona, Spain; 3 Department of Pathology, Hospital Clínic de Barcelona, Universitat de Barcelona, Barcelona, Spain; 4 Department of Infectious Diseases Hospital Clínic de Barcelona, Universitat de Barcelona, Barcelona, Spain; 5 Institut d'Investigacions Biomèdiques August Pi i Sunyer (IDIBAPS), Barcelona, Spain

## Abstract

**Introduction**: Periprosthetic joint infection (PJI) after hip revision surgery shows variable failure rates, with the impact of osteomyelitis in the surrounding bone on outcomes remaining unclear. This study aims to examine bone osteomyelitis prevalence and its impact on hip PJI revision outcomes. **Material and methods**: This retrospective study reviewed 75 cases of chronic hip PJI patients undergoing hip revisions performed at a single center between January 2019 and June 2023. Only cases with peri-implant bone samples submitted for histology evaluation were included. Bone samples were assessed for osteomyelitis using histological criteria. Risk factors, including demographic data, preoperative infections, and previous revisions, were analyzed. Statistical significance was determined using Chi-square and Kaplan–Meier survival analysis ( p≤0.05 ). **Results**: A total of 52 cases of chronic hip PJI were included for final analysis. Up to 30.8 % of the cases ( n=16 ) presented histological signs of osteomyelitis. The success rate among those 36 cases where no signs of osteomyelitis were observed was 88.9 %, whereas, in the 16 cases where it was present, the rate dropped to 37.5 %. Histological signs of osteomyelitis were significantly associated with a prior history of multiple surgeries and unsuccessful antibiotic treatments ( p=0.01 ), the presence of a sinus tract ( p=0.01 ), and the need for additional debridement with spacer exchange after the first stage of a two-stage revision ( p=0.001 ). **Conclusion**: Patients with signs of osteomyelitis demonstrated a higher failure rate. Histological evaluation of periprosthetic bone should ideally be performed during the first stage of revision surgery to guide second-stage management and to improve outcomes.

## 1 Introduction

The failure rates following hip revision resulting from chronic periprosthetic joint infection (PJI) are variable according to the literature, with reinfection rates widely ranging from 0 % to 65 % (Hoberg et al., 2016; Rowan et al., 2018). Several factors have been associated with failure, including those related to the host (Muñoz-Mahamud et al., 2021), the pathogen causing the infection (Dinh et al., 2024; Lora-Tamayo et al., 2013), and the viability of soft and bone tissue. Some cases of chronic PJI present with nonviable tissue, including necrotic bone fragments where bacteria can survive protected from the exposure to systemic antibiotics. It has been reported that complex chronic PJIs require significant bone resection to achieve infection control (Abdelaziz et al., 2021); however, the prevalence and clinical impact of this bone involvement in chronic septic hip revision remains poorly defined. Our hypothesis is that the prevalence of osteomyelitis in septic hip revision surgery is underestimated, and it can be an important factor associated with worse outcomes.

The primary objective of this study was to investigate the prevalence of histological signs of osteomyelitis in prosthetic hip septic revisions, to describe the characteristics of patients with osteomyelitis, and to evaluate its potential impact on the failure rates.

## 2 Material and methods

This was a single-center retrospective review of a prospectively recruited cohort including all hip septic revisions that were performed in our hospital from January 2019 to June 2023 ( n=75 ). For the present study, only patients with a preoperative diagnosis of chronic PJI were included. We excluded those patients who were lost to follow-up during the first 12 months after the revision surgery and patients in which the bone sample for analysis was not obtained. The Institutional Review Board approved the study (register no. HCB/2023/0902).

In all cases, the preoperative study started with a comprehensive physical examination and plain X-rays. Preoperative quantification of C-reactive protein (CRP) and erythrocyte sedimentation rate (ESR) was routinely performed. Regardless of whether any of these tests suspected infection, synovial fluid was aspirated by a percutaneous puncture guided by computerized tomography and submitted for cultures (as well as for white blood cell count in those cases in which enough volume was obtained). The data recorded included the featured demographics (age and sex), American Society of Anesthesiologists score (ASA), presence of a sinus tract, current prolonged unsuccessful antibiotic treatment (defined as more than 4 weeks of antibiotic therapy with evidence of persistent or uncontrolled infection), microbiological results, synovial fluid laboratory findings, and histology of both the periprosthetic membrane and bone, as well as whether the patient had undergone at least two previous surgical procedures related to the infection within the 6 months prior to the revision. Failure was defined as the need for re-revision due to any cause and/or the need for antibiotic suppressive therapy.

All cases that underwent one-stage revision were diagnosed and subsequently treated at our center. The cohort of cases that underwent two-stage revision included both those treated at our institution and others referred from external centers following the failure of an initial medical or surgical approach. From the inception of the study, bone samples for histological analysis were systematically collected in all cases of prosthetic revision due to infection. These samples were subsequently reviewed retrospectively as part of the present study. Cases undergoing two-stage revision without bone samples from the first stage correspond to patients whose initial procedure had been performed at other institutions or before the implementation of systematic bone sampling in our center. All surgical interventions were done by surgeons specifically specializing in revision arthroplasties in a laminar airflow equipped operating theater. Antibiotic prophylaxis was administered at anaesthetic induction, prior to the start of surgery, and was maintained intravenously until adapted according to intraoperative culture results. In patients carrying an antibiotic spacer, the prosthesis-free interval was measured in those undergoing spacer removal, either for spacer exchange or for definitive reimplantation. This interval was defined as the time elapsed between removal of the infected prosthesis (first stage) and reimplantation of the new implant (second stage) or between spacer implantation and spacer exchange in cases where infection control was not achieved after the first stage.

**Figure 1:**
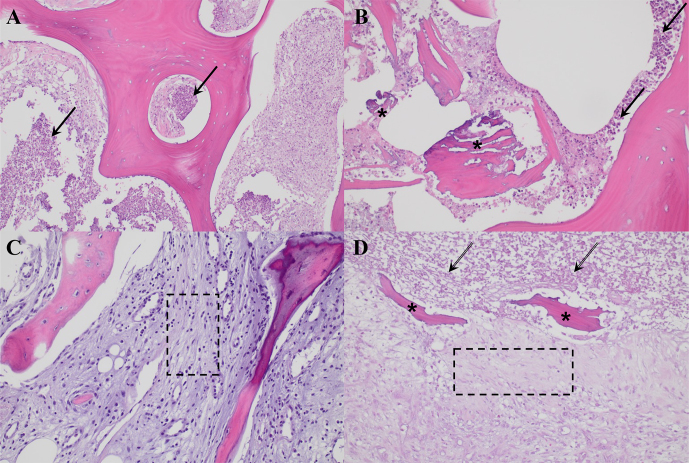
Histological images corresponding to two representative cases of osteomyelitis, as presented in Table 2. Panels **(A)** and **(B)** correspond to case 5, illustrative of a more acute presentation, while panels **(C)** and **(D)** represent case 8, indicative of a more chronic process. **(A)** Histological section (H&E, ×200 ) showing lamellar bone infiltrated by dense neutrophil infiltration (black arrows). **(B)** High-power view (H&E, ×400 ) revealing an area of necrotic bone (asterisks) surrounded by abundant neutrophils (black arrows) and cellular debris. **(C)** Histological section (H&E, ×200 ) showing trabecular bone displaying reactive changes, with bone marrow exhibiting fibrosis and chronic inflammatory infiltrates (dashed-line box). **(D)** Histological section (H&E, ×200 ) showing necrotic bone (asterisks) surrounded by fibrosis and inflammatory cells (dashed-line box) and cellular debris (dashed arrows).

In all cases, synovial fluid was aspirated and sent to the laboratory for white blood cell count, neutrophil count, and microbiological analysis. Our microbiological protocol for culture sample collection features two synovial fluid samples (routinely inoculated into blood culture flasks) and two tissue samples from the neo-synovium, as well as two tissue samples from the interface membrane. In addition, two interface membrane samples were submitted for histology in accordance with the adaption by Feldman and Mirra's criteria (Feldman et al., 1995; Mirra et al., 1976), and at least one bone sample for histological analysis was ground from either bone and/or acetabulum according to the surgeon's own judgment based on both macroscopic findings and complementary tools when available, for instance, preoperative imaging (computerized tomography, magnetic resonance, or single-photon emission computerized tomography) or bone autofluorescence detection under ultraviolet light (Lew and Waldvogel, 2004; Urish and Cassat, 2020). Microscopically, osteomyelitis was defined by the presence of acute and/or chronic inflammatory cells, with or without associated bone necrosis (Fig. 1). The definitive diagnosis of PJI was established according to the criteria defined according to European Bone and Joint Infection Society (EBJIS) criteria (McNally et al., 2021).

Continuous variables reported are the median and interquartile range (IQR). Categorical variables reported are the total number and percentage (%) and were compared using the Chi-square test or Fisher's exact test. Non-parametric continuous variables were compared using the Mann–Whitney U test. A two-sided p≤0.05 was considered to be statistically significant. The log-rank test was used to compare the cumulative probability of revision according to the results of bone histology and time from revision to any-cause failure. Kaplan–Meier curves were plotted with 95 % confidence intervals. Analyses were performed in the SPSS^®^ v. 30.0 statistical package (SPSS, Inc. Chicago, Il, USA).

## 3 Results

Out of the 75 septic hip revisions performed during the study period, a total of 23 cases were excluded due to the lack of histological bone samples for analysis. Finally, a total of 52 cases undergoing hip septic revision procedures were included for the final analysis. The median age was 64.0 years, including 20 women and 32 men. A total of 16 cases (30.8 %) involved one-stage septic revision, 15 cases (28.8 %) involved the first stage of a two-stage revision, and 21 cases (40.4 %) involved the second stage of a two-stage revision (Table 1). A total of 11 cases (21.2 %) presented with a sinus tract at the time of surgery; among these, nine underwent the first stage of a two-stage revision, while only two underwent a single-stage revision. Out of the 52 analyzed procedures, 16 (30.8 %) presented histological signs of osteomyelitis (Table 2).

**Table 1:** Main demographics of the patients included for the final analysis, according to the presence of histological signs of osteomyelitis. SD: standard deviation; CNS: coagulase-negative staphylococci; GNB: gram-negative bacilli; ASA: American Society of Anesthesiologists; BMI: body mass index; PJI: prosthetic joint infection.

Characteristics	Presence of osteomyelitis			p value
	All cases ( n=52 , 100 %)	Yes ( n=16 , 30.8 %)	No ( n=36 , 69.2 %)	
Median (IQR) age	64.0 (49.2–75.0)	60.0 (38.0–68.2)	66.0 (55.0–79.2)	
Sex (%) Male Female	32 (61.5 %) 20 (38.5 %)	9 (56.3 %) 7 (43.8 %)	23 (63.9 %) 13 (36.1 %)	0.75
ASA score (%) I-II III-IV	26 (50.0) 26 (50.0)	9 (56.3 %) 7 (43.8 %)	17 (44.4 %) 19 (47.2 %)	0.79
Median (IQR) BMI	27.7 (24.2–31.6)	29.5 (23.8–37.3)	26.3 (24.2–31.1)	
Type of revision (%) One-stage Two-stage First stage Second stage	16 (30.8 %) 15 (28.8 %) 21 (40.4 %)	1 (6.3 %) 13 (81.3 %) 2 (12.5)	15 (41.7 %) 2 (5.6 %) 19 (52.8 %)	0.01
Laboratory values Median (IQR) CRP CRP > 1 mg dL^−1^ Median (IQR) ESR	2.1 (0.8–4.4) 35 (67.3 %) 27.0 (9.5–65.0)	4.3 (1.5–13.0) 13 (81.3 %) 66 (35.0–83.0)	1.2 (0.5–3.2) 22 (61.1 %) 20 (7.5–48.0)	0.17
ESR > 30 mm h^−1^	19 (36.5 %)	9 (56.3 %)	10 (27.8 %)	0.01
Microorganism (%)^*^ CNS *Staphylococcus epidermidis* *Staphylococcus lugdunensis* Other CNS *Staphylococcus aureus* *Streptococcus *spp. *Cutibacterium acne* *Proteus mirabilis* *Escherichia coli* *Pseudomonas aeruginosa* *Enterococcus* spp. *Bacillus* spp. *Candida *spp. *Micrococcus* spp. *Prevotella *spp. Negative cultures	17 (32.7 %) 13 (25.0 %) 1 (1.9 %) 3 (5.8 %) 3 (5.8 %) 3 (5.8 %) 3 (5.8 %) 2 (3.8 %) 3 (5.8 %) 1 (1.9 %) 3 (5.8 %) 1 (1.9 %) 2 (3.8 %) 1 (1.9 %) 1 (1.9 %) 16 (30.8 %)	4 (25.0 %) 3 (18.7 %) 0 1 (6.2 %) 1 (6.2 %) 1 (6.2 %) 2 (12.5 %) 1 (6.2 %) 2 (12.5 %) 1 (6.2 %) 3 (18.7 %) 0 1 (6.2 %) 0 0 2 (12.5 %)	13 (36.1 %) 10 (27.8 %) 1 (2.8 %) 2 (5.5 %) 2 (5.5 %) 2 (5.5 %) 1 (2.8 %) 1 (2.8 %) 1 (2.8 %) 0 0 1 (2.8 %) 1 (2.8 %) 1 (2.8 %) 1 (2.8 %) 14 (38.9)	
Polymicrobial infection (%)	11 (21.2 %)	4 (25.0 %)	7 (19.4)	0.07
Sinus tract (%)	11 (21.2 %)	9 (56.3 %)	2 (5.6)	0.01
Any-cause failure (%) Postoperative acute PJI Persistence of infection Suppressive antibiotic Death related to PJI	14 (26.9 %) 2 (3.8 %) 9 (17.3 %) 2 (3.8 %) 1 (1.9 %)	10 (62.5 %) 1 (6.3 %) 8 (50.0 %) 0 1 (6.3 %)	4 (11.1) 1 (2.8 %) 1 (2.8 %) 2 (5.6 %) 0	0.01
Under long antibiotic treatment	20 (38.5 %)	13 (81.3 %)	7 (19.4 %)	0.01

^*^ Microorganisms isolated in cultures from samples obtained during the index surgery for analysis.

**Table 2:** Summary of the 16 cases exhibiting histological signs of osteomyelitis. S1: first stage of a two-stage revision; S2: second stage of a two-stage revision; 1SR: single-stage revision; BMI: body mass index; CNS: coagulase-negative *staphylococci*; SAUR: *Staphylococcus aureus*; PMN: polymorphonuclear leukocytes; PAI: postoperative acute infection; PI: persistence of infection; MRSA: methicillin-resistant *Staphylococcus aureus*; ATB: antibiotic.

n	Sex	Age	BMI	Type of	Microorganism^a^	Under prior failed	Sinus	Membrane	Failure	Bone histological			
				surgery		ATB treatment	tract	PMN		findings			
										Neutrophil	Chronic inflammatory	Necrotic	Marrow
										infiltration	cells	bone	fibrosis
1	Female	66	23.0	S1	MRSA	Yes	Yes	Negative	PI	Yes	Yes	No	Yes
2	Male	40	44.0	S1	*E. faecalis* CNS	Yes	Yes	> 5	PI	No	Yes	Yes	Yes
3	Male	44	42.7	S1	*K. pneumoniae* *S. haemolyticus* *S. aureus*	Yes	Yes	> 5	PI	Yes	Yes	No	Yes
4	Female	69	28.3	S1	*E. coli*	Yes	No	> 5	PI	No	Yes	No	Yes
5	Male	34	29.2	S1	*Klebsiella pneumoniae*	No	No	Negative	PI	Yes	No	Yes	No
6	Male	79	29.9	S1	*E. coli* *Enterococcus* spp.	Yes	Yes	> 5	Death^b^	Yes	Yes	No	Yes
7	Female	64	30.8	S1	*C. acnes*	Yes	No	> 5	No	Yes	No	No	Yes
8	Male	61	33.9	S1	*K. pneumoniae*	Yes	No	> 5	PI	No	Yes	Yes	Yes
9	Male	75	26.4	S1	*E. faecalis*	Yes	Yes	Negative	PI	No	Yes	Yes	Yes
10	Female	42	38.4	S1	*Proteus mirabilis*	Yes	Yes	> 5	PI	Yes	Yes	Yes	Yes
11	Female	42	38.4	S2	Negative	Yes	No	Negative	PAI	Yes	Yes	Yes	Yes
12	Male	59	29.0	1SR	*S. epidermidis* *S. capitis*	No	No	> 5	No	No	Yes	Yes	Yes
13	Female	61	31.6	S2	*Cutibacterium acnes*	No	Yes	> 5	No	Yes	No	Yes	No
14	Male	29	22.9	S1	*Enterobacter cloacae*	Yes	Yes	Negative	No	No	Yes	No	Yes
15	Male	29	22.9	S2	Negative	Yes	No	Negative	No	No	Yes	Yes	Yes
16	Female	80	21.1	S1	*Candida albicans*	Yes	Yes	> 5	No	Yes	No	Yes	No

^a^ Microorganisms isolated from intraoperative samples and/or synovial fluid obtained through preoperative percutaneous aspiration. ^b^ Death related to PJI.

We identified three main clinical characteristics associated with the presence of histological signs of osteomyelitis. First was those cases in which the patient had undergone multiple prior surgeries and experienced prolonged unsuccessful antibiotic treatments ( p=0.01 ). Aside from this, all of these cases were also associated with higher levels of acute-phase reactants, primarily driven by an elevation in the ESR. Second was the presence of a sinus tract. Among the 11 cases with a sinus tract, only 2 belonged to the group that underwent a one-stage revision, while the remaining 9 were part of the group in which the first stage of a two-stage revision was performed ( p=0.01 ). Finally, the strongest association was observed in cases involving the first stage of a two-stage septic revision that failed due to persistent infection and that required additional debridement with spacer exchange ( p=0.001 ).

**Figure 2:**
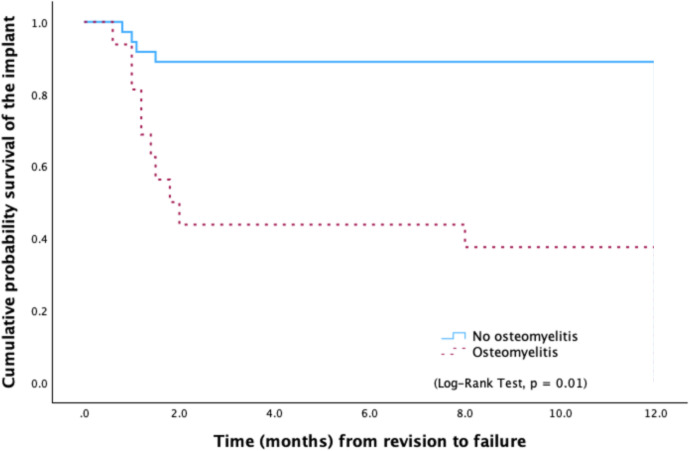
Kaplan–Meier survival curve showing significant differences in the probability of any-cause failure according to the presence of histological signs of osteomyelitis.

In cases where no signs of osteomyelitis were observed, the 1-year failure rate was 11.1 %. Conversely, when osteomyelitis was present, the failure rate increased markedly to 62.5 %, primarily due to the need for additional debridement and spacer exchange (Fig. 2).

In cases requiring a spacer exchange, the median (IQR) interval between surgeries was 3.2 weeks (2.0–9.0). The median (IQR) time between first-stage surgery and reimplantation was 27.0 weeks (10.1–57.6). Although not statistically significant ( p=0.09 ), a trend toward shorter intervals was observed in patients with osteomyelitis.

## 4 Discussion

The success rate of septic revision, whether conducted in one or two stages, typically falls within the range of approximately 80 % to 95 % according to various series (Akgün et al., 2018; Hoberg at al., 2016; Kunutsor et al., 2018). It has been proposed that a plausible cause of persistent infection after septic hip revision may stem from the presence of non-viable bone in the proximal femur, in contact with the articular cavity.

In certain cases, implant-associated osteomyelitis may lead not only to the destruction of the implant cavity contour but also to the development of an avascular zone of necrotic bone tissue and associated hypoxia. Such local tissue alterations may hinder both antibiotic penetration and oxygen delivery (Jensen et al., 2017). Nonetheless, histological findings are often non-pathognomonic and do not allow for a definitive distinction between bone infection and inflammation. Our observations most likely reflect the involvement of the adjacent bone in periprosthetic infection, where both osteomyelitis and osteitis processes may coexist to varying degrees. Moreover, the inflammatory cells observed in PJI do not originate solely from the joint or the implant itself but may migrate from the peripheral circulation and surrounding tissues and can therefore be consistently identified within the bone adjacent to an infected implant (Biedermann et al., 2023). Consequently, the findings probably represent a continuum of inflammatory and infectious changes extending into the cortical bone surrounding the implant or the implant cavity, making the strict use of either term (osteitis or osteomyelitis) challenging in certain cases. It must also be acknowledged that repeated surgical interventions may contribute to the inflammatory changes observed in the surrounding bone. In the present series, a tendency toward more pronounced bone inflammation was noted in cases with a recent surgical procedure, suggesting that, in some instances, these findings might represent a reactive postoperative process rather than persistent infection.

The present series highlights that those cases without histological evidence of osteomyelitis achieved a success rate of 88.9 %, whereas this rate decreased significantly to 37.5 % in those cases with confirmed osteomyelitis. Abdelaziz et al. reported a series of 57 one-stage septic revisions in which proximal femoral resection was frequently required to reduce the risk of recurrent infection (Abdelaziz et al., 2021). This decision, however, is often made intraoperatively based on direct surgical findings, underlining how identifying non-viable bone can be challenging yet essential for infection control. Beyond biofilm on the implant surface, residual bone infection has also been proposed as a potential cause of failure in patients under suppressive antibiotic therapy (Escudero-Sanchez et al., 2020).

**Figure 3:**
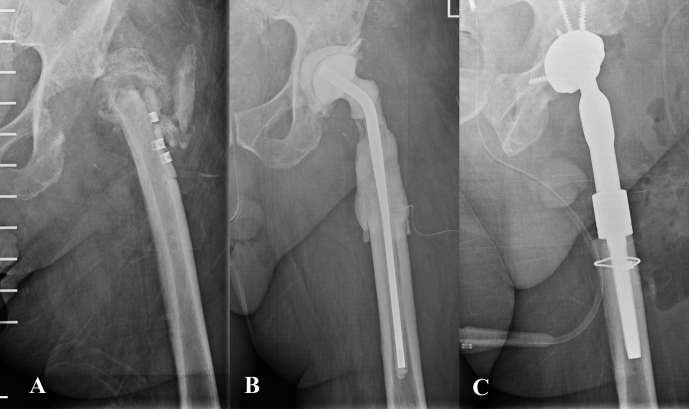
**(A)** Anteroposterior pelvic radiograph of a patient with chronic prosthetic joint infection of the left hip. The patient had previously undergone multiple procedures involving osteosynthesis material. Both the patient's medical history and the diffuse radiographic appearance of the proximal femur suggested bone involvement compatible with osteomyelitis. **(B)** A wide resection of the proximal femur was undertaken, and a preformed spacer was implanted. Histological analysis revealed mild neutrophil infiltration and chronic inflammatory changes within trabecular bone and bone marrow, consistent with osteomyelitis. **(C)** Hip reconstruction was subsequently performed using a megaprosthesis owing to the substantial bone loss in the proximal femur. This procedure may also be undertaken in a one-stage approach, although accurately determining the true extent of femoral bone involvement can be challenging.

In our cohort, histological signs of osteomyelitis were more frequently observed in cases with multiple prior surgeries, sinus tract formation, or prolonged unsuccessful antibiotic therapy, suggesting a higher degree of chronicity. In line with our results, Abdelaziz et al. (2021) identified the presence of a sinus tract, longer duration from primary implantation to index infection, and multiple prior revisions as predictors of proximal resection due to non-viable bone. In our retrospective series, histological bone samples were obtained in more than half of the patients; however, in some cases, limited bone stock hindered adequate sampling, potentially compromising implant stability. In such situations, surgeons may opt for a targeted biopsy of bone suspected to be affected by osteomyelitis, aiming to improve diagnostic accuracy and to guide resection. However, as osteomyelitis can be irregularly distributed (Sigmund et al., 2023), undetected infected areas may persist despite targeted sampling. Based on these considerations, the bone sampling strategy becomes a key component of the diagnostic process. Obtaining multiple bone samples (ideally three to six) during the first-stage procedure, when active infection is most likely, is recommended (McNally et al., 2021; Sigmund et al., 2023). This approach increases the likelihood of detecting infection that may otherwise be missed with limited or localized sampling, thereby improving diagnostic accuracy. Moreover, adequate histological information from the first stage can guide more precise bone resection and reconstruction decisions at reimplantation, potentially reducing residual infection and improving outcomes.

It remains crucial to acknowledge the diagnostic challenges associated with persistent bone infection. While osteitis refers to bone inflammation without necessarily implying infection, osteomyelitis may lack specific features that clearly distinguish it from other inflammatory or reactive processes, particularly in limited tissue samples. Correlation with the clinical course and complementary investigations are therefore essential (Muñoz-Mahamud et al., 2021). At the time of reimplantation, inflammatory changes observed in bone samples may reflect either residual infection or a reparative response to the first-stage surgery. Differentiating between these entities based solely on histology is often difficult and should rely on integration with intraoperative assessment and microbiological findings. However, in the present series, bone samples obtained for histological evaluation were not systematically submitted for microbiological culture. In some cases, the limited amount of bone and the need to preserve stock prevented additional sampling. Performing parallel histological and microbiological assessment could provide complementary information and may help better characterize bone involvement in future studies.

**Figure 4:**
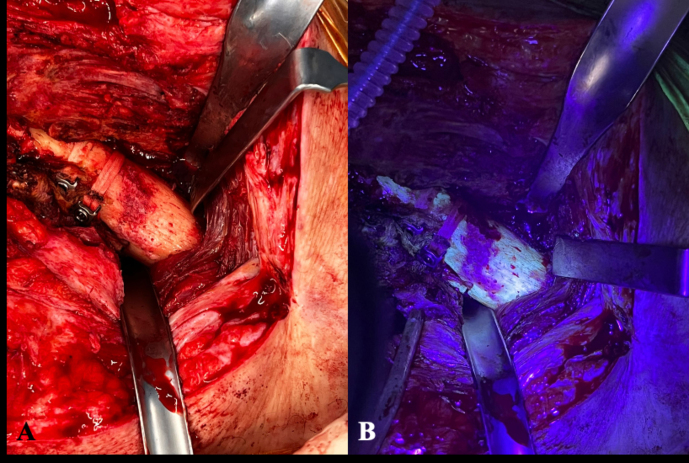
**(A)** Intraoperative clinical photographs showing the anterior aspect of the proximal femur of a patient operated on using an anterolateral approach. The picture exemplifies the usual difficulty in distinguishing viable from necrotic bone. **(B)** The surgical field after the utilization of fluorescent light as an example of an intraoperative tool to aid in bone sample collection. Under the black light, viable metaphyseal bone is supposed to glow greenish, whereas all of that bone failing to fluoresce may be considered to be necrotic.

Surgical excision of non-viable bone is often challenging due to the difficulty in distinguishing necrotic from viable tissue. Few tools are available to assist surgeons during this process, and bone appearance remains somewhat subjective and dependent on the surgeon's judgment (Abdelaziz et al., 2021; Lew and Waldvogel, 2024; Rupp et al., 2020). Alternative techniques have been proposed to visualize infected tissue, such as laser Doppler flowmetry or intra-articular methylene blue (Duwelius and Schmidt, 1992; Rosenberg and Khurana, 2016). In our experience, tetracycline bone labeling combined with Wood's lamp detection of non-viable bone provided a visual contrast between viable and necrotic areas under ultraviolet light (McPherson et al., 2002). In the present series, none of the patients were undergoing tetracycline treatment at the time of bone sample collection; however, when available, Wood's lamp was used intraoperatively to guide sampling (Fig. 4) as some tissues have been reported to exhibit autofluorescence under UV illumination (Hoell et al., 2006; Rowan et al., 2018; Swiontkowski, 1990; Yoshiga et al., 2015). Other studies, however, have found that autofluorescence-guided sampling does not appear to be superior to conventional techniques (Giudice et al., 2018). Hence, the efficacy of UV illumination without prior tetracycline labeling remains debatable.

Limitations of the study include its retrospective design and potential biases. The series comprises heterogeneous cases of septic revision, with the choice between one-stage and two-stage strategies being subjectively determined by the surgeon based on experience and preoperative tests. Accurately determining the exact number of previous surgeries and the precise duration of prior antibiotic treatments proved to be challenging, particularly in patients referred from other centers, where medical records were often incomplete and where treatment regimens were inconsistently administered over extended periods. These factors inevitably limit the strength of the conclusions that can be drawn from the present study. Another limitation of the study is the loss of patients undergoing revision surgery where bone samples for analysis were not obtained. It is noteworthy that the majority of these cases involved one-stage revisions where bone preservation is highly prioritized, making it so that it is not always straightforward to obtain bone samples. Furthermore, bone samples obtained for histological analysis were not systematically submitted for microbiological culture as, in several cases, the limited bone stock precluded additional sampling. Incorporating both histological and microbiological assessment could have provided complementary information and should be considered in future studies. Finally, a key limitation of the present study concerns the method of bone sampling. In each case, the sample was obtained from the site the surgeon considered to be most appropriate, primarily based on intraoperative judgment. A major drawback lies in the fact that histological analysis was performed on a single bone specimen, typically from the femur and, in some instances, the acetabulum. Given that osteomyelitis is not uniformly distributed within bone, there is a risk of overlooking focal areas of infection. Previous studies, including that of Sigmund et al., have demonstrated that multiple samples significantly improve diagnostic accuracy (Sigmund et al., 2023). It is therefore possible that a more extensive sampling protocol might have identified histological signs of infection in all cases, potentially influencing the interpretation of our results. In this regard, a standardized approach involving multiple samples from defined areas of both the femur and acetabulum should be considered. Prospective studies with larger cohorts are needed to clarify the true impact of osteomyelitis on the outcome of septic hip revision.

## 5 Conclusions

In conclusion, patients with signs of osteomyelitis demonstrated a higher failure rate. Histological evaluation of periprosthetic bone should ideally be performed during the first stage of revision surgery to guide second-stage management and to improve outcomes. The presence of osteomyelitis was predominantly identified in cases characterized by the presence of a sinus tract, a history of multiples surgeries followed by prolonged unsuccessful antibiotic treatments, and subsequent failure requiring additional debridement and spacer exchange.

## Data Availability

Raw data can be made accessible upon reasonable request to the corresponding author (Ernesto Muñoz-Mahamud: emunoz@clinic.cat).
